# Evaluation of Dietary Soluble Extract Hydrolysates with or without Supplementation of Inosine Monophosphate Based on Growth, Hematology, Non-Specific Immune Responses and Disease Resistance in Juvenile Nile Tilapia *Oreochromis niloticus*

**DOI:** 10.3390/ani11041107

**Published:** 2021-04-12

**Authors:** Jinho Bae, Yujin Song, Mohammad Moniruzzaman, Ali Hamidoghli, Seunghan Lee, Hyeongwoo Je, Wonsuk Choi, Taesun Min, Sungchul C. Bai

**Affiliations:** 1 Feeds & Foods Nutrition Research Center, Pukyong National University, Busan 48513, Korea; bjh2921@naver.com (J.B.); ssongyou92@naver.com (Y.S.); ali.hamidoghli@yahoo.com (A.H.); jhw245@gmail.com (H.J.); thm622@naver.com (W.C.); 2 Department of Animal Biotechnology, Jeju International Animal Research Center (JIA) & Sustainable Agriculture Research Institute (SARI), Jeju National University, Jeju 63243, Korea; monir1983@jejunu.ac.kr (M.M.); tsmin@jejunu.ac.kr (T.M.); 3 Aquafeed Research Center, National Institute of Fisheries Science, Pohang 37517, Korea; seunghanlee@korea.kr; 4 FAO World Fisheries University Pilot Program/Feeds and Foods Nutrition Research Center (FFNRC), Pukyong National University, 365, Sinseon-ro, Nam-gu, Busan 48547, Korea

**Keywords:** fishery by-products, inosine monophosphate, growth performance, hematology, non-specific immune responses, disease resistance, Nile tilapia

## Abstract

**Simple Summary:**

This study was conducted to elucidate the effects of dietary soluble extract hydrolysates obtained from fishery by-products, such as shrimp soluble extract (SSE), tilapia soluble extract (TSE) and squid soluble extract (SQSE). Furthermore, we used a nucleotide, inosine monophosphate (IMP), as an additive in different concentrations along with shrimp soluble extract to understand their effects on growth, immunity and disease resistance in juvenile Nile tilapia. Our results demonstrated that dietary SSE could improve growth performance, non-specific immune responses and disease resistance against pathogenic bacteria *Aeromonas hydrophila* in juvenile Nile tilapia. Moreover, IMP did not add further benefits to the SSE diet. Further research is needed to better understand the effects of fishery by-products and IMP on fish diets.

**Abstract:**

We performed an 8-week feeding trial to evaluate dietary soluble extract hydrolysates from fishery by-products, such as shrimp soluble extract (SSE) with or without inosine monophosphate (IMP), tilapia soluble extract (TSE) and squid soluble extract (SQSE), in juvenile Nile tilapia. A diet without feed additives was used as the control diet (CON); and five other experimental diets were formulated with 2% soluble extracts consisting of 100% SSE, 98% SSE + 2% IMP (SSEP_2_), 96% SSE + 4% IMP (SSEP_4_), 100% SQSE and 100% TSE. The diets were fed to 4.9 ± 0.07 g (mean ± SD) juvenile Nile tilapia in triplicate groups. The weight gain and specific growth rates of fish fed the SSE, SSEP_2_ and SSEP_4_ diets were significantly higher than those of fish fed the CON and SQSE diets. The superoxide dismutase activity levels of fish fed the SSE and SSEP_4_ diets were significantly higher than those of fish fed the CON, SSEP_2_, SQSE and TSE diets. Myeloperoxidase activity levels of fish fed the SSE and SSEP_4_ diets were significantly higher than those of fish fed the CON, SSEP_2_ and SQSE diets. Lysozyme activity levels of fish fed the SSEP_4_ and SQSE diets were significantly higher than those of fish fed the SSE and SSEP_2_ diets. Feed efficiency, protein efficiency ratio, survival rate, whole body proximate composition and hematological parameters were not significantly different among the groups. After ten days of challenge = against *Aeromonas hydrophila*, the cumulative survival rate of fish fed the SSE diet was significantly higher than those of fish fed the CON, SQSE and TSE diets. In conclusion, dietary shrimp soluble extract could improve the growth performance, non-specific immune responses and disease resistance in juvenile Nile tilapia, and inosine monophosphate did not add further benefits to this ingredient.

## 1. Introduction

Aquaculture is considered as one of the fastest growing food producing industries, which supplies over 50% of global fish production [1]. However, the future development of aquaculture is limited by the excessive use of unsustainable marine ingredients in aquafeed. A significant number of research studies have been conducted on alternative protein sources, mostly with plant origins [2,3,4,5]. However, plant ingredients often lack the essential amino acids for fish and/or possess low digestibility and palatability [6]. Fishery by-products which contain essential nutrients can be utilized as useful ingredients in aquafeed production [7]. According to previous studies, protein hydrolysates derived from fishery by-products have been considered as beneficial ingredients in feed for fish because of their nutritional, functional and cost-effective properties [8].

Hydrolysis of feed ingredients through the enzyme processes has been used as one of the important methods for the processing of fishery by-products [8,9]. This processing method results in small molecular weight compounds and hydrolysates with considerably diverse amino acid profiles—potential feed additives and fishmeal replacers for aquaculture [10]. Crustacean hydrolysates, such as shrimp soluble extract (SSE), have been used in aquafeed as protein sources [11,12] and as dietary supplements for improvements to palatability and acceptability for fish [13]. Likewise, hydrolysates of fishery by-products such as tilapia soluble extract (TSE) and squid soluble extract (SQSE) have essential amino acids that are required for the fish growth and survival, and have positive effects, such as enhancing immune response and palatability [14,15,16,17]. SSE, TSE and SQSE contain high levels of amino acids, especially in free form, and active peptides that can be digested and assimilated by aquatic organisms [18,19]. Furthermore, the use of SSE, TSE and SQSE could reduce environmental problems by processing and using the inedible parts of fishery products in the diets of cultured fish.

Nile tilapia, *Oreochromis niloticus*, is one of the extensively cultured fish species with high economic importance for the aquaculture industry because of its faster growth, higher survivability in high stocking densities and higher disease resistance compared to the other freshwater fish species [20]. Nile tilapia has become an iconic freshwater-cultured fish species which contributed about 4.5 million tons to the market in 2018 based on the global aquaculture production report by FAO [1]. However, with the expansion of intensive aquaculture, tilapia farms have been more susceptible to disease outbreaks. For example, *Aeromonas hydrophila* is a bacterium that has caused massive rates of mortality in tilapia farms around the world [21]. Additionally, due to the use of low marine ingredients (e.g., fishmeal) in the diet of this species, the growth, feed conversion ratio (FCR) and protein efficiency ratio in fish are often affected negatively [21,22,23]. These negative effects of feed ingredients in fish may occur due to lower palatability and/or absence of key compounds such as nucleotides [24]. Inosine monophosphate (IMP), a key compound in purine nucleotide metabolism, has been shown to benefit physiological and nutritional functions in different animals as a dietary supplement [25,26]. According to previous research results, the growth performance, feed intake, immune responses and disease resistance were significantly improved in fish when IMP was used alone or combined with some free amino acids in the diet [27,28,29,30]. The non-specific immune response in terms of lysozyme activity, myloperoxidase and nitro-blue-tetrazolium activities was improved with dietary supplementation of IMP in olive flounder (*Paralichthys olivaceus*) [28]. In Nile tilapia, final body weight, growth-related gene expression, feed utilization performance and immune responses have been improved by the addition of IMP in the diet [29,30].

In our previous studies, Moniruzzaman et al. [2] and Jo et al. [12] reported that dietary supplementation of SSE has positive effects on growth and immune responses in freshwater fish such as rainbow trout. Based on the previous findings, the aim of the present study was to determine the effects of dietary soluble extract hydrolysates from fishery by-product, such as shrimp soluble extract (SSE), with or without the addition of inosine monophosphate (IMP), in comparison to the diets supplemented with tilapia soluble extract (TSE) and squid soluble extract (SQSE) in terms of growth performance, hematology, innate immune responses and disease resistance in juvenile Nile tilapia *Oreochromis niloticus*.

## 2. Materials and Methods

### 2.1. Experimental Diets

Fishery by-products, such as shrimp soluble extract, squid soluble extract and tilapia soluble extract, were provided by VNF Company (Vietnam Food Joint Stock Company, Ho Chi Minh City, Vietnam). The fishery by-product production process goes through several steps to eliminate extraneous matter, and then they are shredded and pressed to obtain a liquid extract. Later, this liquid extract went through a centrifugal process to acquire its purest form. The extract is then sent to chemical processing area where the protein is broken into peptides and digestible amino acids. Chemically processed extract is refined to create products that will meet various levels of quality standards according to customer demands. Refined soluble is mixed and added with flavor-preservation additives, to maintain the product’s unique flavor.

Six experimental diets were formulated to have the same crude protein (33%) levels. The ingredients and proximate compositions of the six experimental diets are shown in Table 1. A basal diet without feed additives was used as the control (CON); the other five diets were formulated to include 2% of soluble extracts: 100% shrimp soluble extract (SSE), 98% shrimp soluble extract +2% inosine monophosphate (SSEP_2_), 96% shrimp soluble extract +4% inosine monophosphate (SSEP_4_), 100% squid soluble extract (SQSE) and 100% tilapia soluble extract (TSE), replacing the total of 2% consisting of soybean meal, wheat flour and soybean oil from the CON diet to balance the nutritional compositions of the diets. Fish meal, soybean meal, rapeseed meal, meat and bone meal, poultry by-product meal and squid liver powder were used as the protein sources; soybean oil and fish oil were used as the lipid sources; and wheat flour was used as the carbohydrate source in the experimental diets. The feed preparation procedure was followed described elsewhere by Hamidoghli et al. [31]. Pellets were air-dried for 48–96 h, broken and sieved to get the desired size and stored at −20 °C until use.

### 2.2. Experimental Fish

The juvenile Nile tilapia were obtained from a private hatchery (Docheon Aquafarm, Changnyeong, Korea), and the experiment was conducted at the laboratory facilities of the Feeds and Foods Nutrition Center (FFNRC), Pukyong National University, Busan, Korea. Prior to the execution of experiment, fish were fed a commercial diet for two weeks to be acclimated to the experimental environment. Three hundred sixty fish averaging 4.9 ± 0.07 g (mean ± SD) were randomly distributed into 18 tanks (20 fish/tank) of 30 L volume of filtered freshwater. Each experimental feed was fed to triplicate groups of fish up to apparent satiation twice daily (09:00 and 18:00 h) for 8 weeks. Water temperature and pH were maintained at 27 ± 0.5 °C and 7.5 ± 0.3 and aeration was supplied for sufficient dissolved oxygen to each tank.

### 2.3. Sample Collection and Analyses

At the end of the 8-week feeding trial, fish were starved for 24 h, and the total number and weight of fish in each tank was determined for calculations of weight gain (WG), specific growth rate (SGR), feed efficiency (FE), protein efficiency ratio (PER) and survival rate. Three fish per tank (nine fish per treatment) were randomly selected and stored at −20 °C for whole-body proximate composition. Three additional fish per tank were randomly sampled, individually weighed and then dissected to obtain liver and viscera samples for the determination of hepatosomatic index (HSI), viscerosomatic index (VSI) and condition factor (CF). Three fish per tank were randomly captured and anesthetized with ethylene glycol phenyl ether (200 ppm) and blood samples were obtained via caudal vein puncture using a non-heparinized 1 mL syringe. Blood samples were allowed to clot at room temperature for 30 min. Then, the serum was separated by centrifugation at 5000 *g* for 10 min and stored at −80 °C for the analysis of non-specific immune responses, including superoxide dismutase (SOD), myeloperoxidase (MPO) and lysozyme activity.

### 2.4. Proximate Composition 

Proximate composition, including moisture, crude protein, crude lipid and ash of the diets and whole-body samples were measured based on AOAC [32]. Briefly, parts of the diets and fish samples were dried at 135 °C for 2 h to obtain the moisture contents. Ash contents were determined by incineration at 550 °C for 3 h in muffle furnace. Crude lipid contents were achieved by Soxhlet extraction process (Soxtec system 1046, Tecator AB, Hoganas, Sweden) using ethyl alcohol as organic solvent, and crude protein contents were analyzed by the Kjeldahl method based on nitrogen concentrations in the samples (Nx6.25) after the digestion, distillation and titration.

### 2.5. Hematological Parameters

Blood plasma glucose (GLU), total cholesterol (T-CHO), aspartate aminotransferase (AST) and alanine aminotransferase (ALT) were measured by a chemical analyzer (Fuji DRI-CHEM3500i, Tokyo, Japan).

### 2.6. Non-Specific Immune Parameters

Serum SOD was measured using an assay kit (Sigma-Aldrich, St. Louis, MI, USA, 19160), following the manufacturer’s guidelines. This method is based on inhibition against WST (Water soluble tetrazolium dye) and determination of SOD enzyme activity. The absorbance was read at 450 nm after incubating samples for 20 min at 37 °C. Lysozyme activity was used for determination of serum lysozyme level by the method described by Hultmark et al. [33] with slight modifications. Lysozyme activity was determined by reaction against *Micrococcus lysodeikticus* and microplate reader (Sunrise TECAN, Männedorf, Switzerland) analysis with 450 nm. Myeloperoxidase (MPO) was measured according to Quade and Roth [34]. Briefly, 20 µL of serum was diluted with Hanks balanced salt solution (HBSS) without Ca^2+^ or Mg^2+^ in 96-well plates. Then, 35 µL o f 3.3′5.5′- tetramethylbenzidine hydrochloride (TMB, 20 mM) (Sigma-Aldrich) and H_2_O_2_ (5 mM) were added. The color change reaction was stopped after 2 min by adding 35 µL of 4 M sulfuric acid. Absorbance was read at 450 nm in the micro-plate reader.

### 2.7. Challenge Test

In the challenge test with *Aeromonas hydrophila*, bacteria were incubated at 27 °C for 24 to 48 h in BHI broth medium and then suspended in sterile distilled water at 1 × 10^7^ CFU/mL from Department of Biotechnology, Pukyong National University (Busan, Korea). The methods for bacteria culture, CFU estimation and concentration adjustment were followed as previously described by Hasan et al. [35]. Fifteen fish (triplicate groups of five fish per tank) were distributed according to their dietary treatment groups into a 50 L tank for the challenge test without water exchange. After injecting 0.1 mL of the suspension into the peritoneal cavity (1 × 10^6^ CFU/ per fish), the survival rate of Nile tilapia was investigated according to elapsed time without feeding, and the experimental group and the control group were compared and analyzed.

### 2.8. Statistical Analysis

After confirming normality and homogeneity of variance using Leven’s Test for equality of variances, data were analyzed by one-way ANOVA using the IBM SPSS 26 statistics program. The least significant difference (LSD) multiple range test was used as a post-hoc method, and the significance level was set at *p* < 0.05. Cumulative survival rate was presented with a Kaplan–Meier plot using the GraphPad Prism 8.4.0 (GraphPad Software Inc., San Diego, CA, USA) application. Survival curves were compared by log-rank (Mantel-Cox) for trend analysis, and Gehan–Breslow–Wilcoxon tests.

## 3. Results

### 3.1. Growth Performance and Survival Rate

After eight weeks of feeding trial, growth performance and survival rates of fish fed the different experimental diets were evaluated (see Table 2). The weight gain (WG) and specific growth rates (SGR) of fish fed SSE, SSEP_2_ and SSEP_4_ diets were significantly higher than those of fish fed CON and SQSE diets (*p* < 0.05). However, there were no significant differences in the WG and SGR of fish fed the SSE, SSEP_2_, SSEP_4_ and TSE diets (*p* > 0.05). Total feed intake (FI) for fish fed the SSE and SSEP_4_ diets was significantly higher than for fish fed the CON diet. However, there were no significant differences in FI for fish fed the SSE, SSEP_2_, SSEP_4_, SQSE and TSE diets (*p* > 0.05). Feed efficiency (FE), protein efficiency ratio (PER) and survival percentage were not significantly different among fish fed any of the experimental diets (*p* > 0.05). Hepatosomatic index (HSI), viscerosomatic index (VSI) and condition factor (CF) showed no significant differences in all the experimental groups (*p* > 0.05).

### 3.2. Whole-Body Proximate Composition

Whole-body proximate compositions of Nile tilapia fed the six experimental diets are shown in Table 3. There were no significant differences in moisture, crude protein, crude lipid or crude ash for fish fed the experimental diets (*p* > 0.05).

### 3.3. Hematological Parameters

The results of hematological parameters are presented in Table 4. There were no significant differences in aspartate aminotransferase (AST), alanine aminotransferase (ALT), glucose (GLU) or total cholesterol (TCHO) contents of fish fed the experimental diets (*p* > 0.05).

### 3.4. Non-Specific Immune Responses

**Figure 1:**
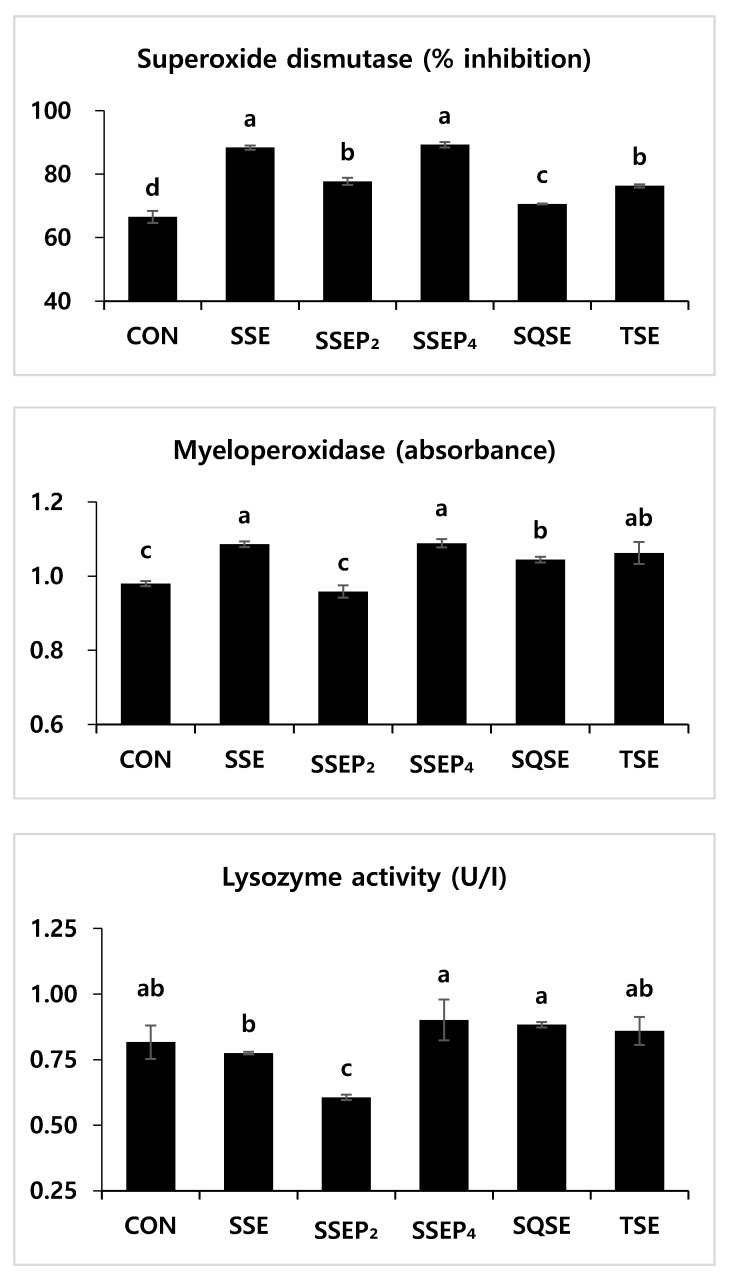
Non-specific immune responses of juvenile Nile tilapia fed six experimental diets in triplicate (*n* = 3) for eight weeks. Values represent mean ± SD (*n* = 3). Different letters (a,b,c) indicate a significant difference (*p* < 0.05).

Non-specific immune responses, including the myeloperoxidase (MPO), superoxide dismutase (SOD) and lysozyme activity of fish fed the six experimental diets, are presented in Figure 1. The SOD activity levels of fish fed SSE and SSEP_4_ diets were significantly higher than those of fish fed CON, SSEP_2_, SQSE and TSE diets (*p* < 0.05). MPO activity levels of fish fed SSE and SSEP_4_ diets were significantly higher than those of fish fed CON, SSEP_2_ and SQSE diets (*p* < 0.05); however, there was no significant differences among the former two and the TSE group (*p* > 0.05). The lysozyme activity levels of fish fed SSEP_4_ and SQSE diets were significantly higher than those of fish fed SSE and SSEP_2_ diets (*p* < 0.05). However, there were no significant differences in the lysozyme activity levels of fish fed SSEP_4_, SQSE, CON and TSE diets (*p* > 0.05).

### 3.5. Bacterial Challenge Test

**Figure 2:**
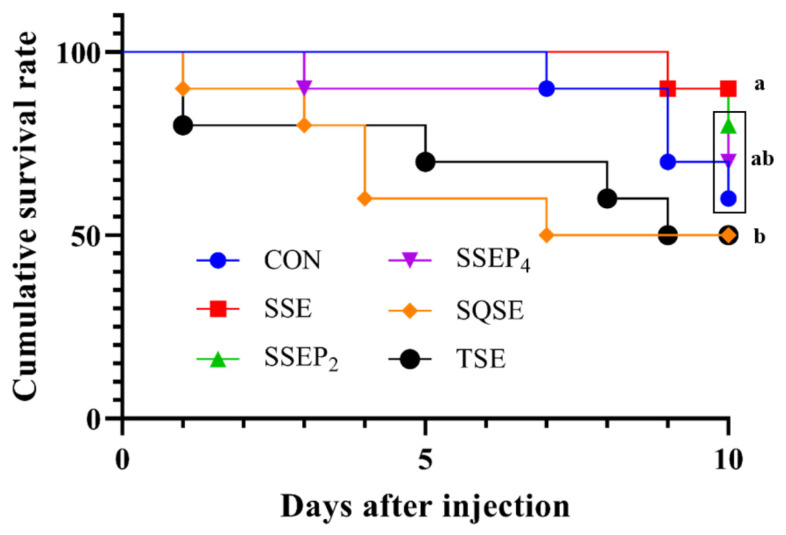
Cumulative survival rate of Nile tilapia fed six experimental diets in triplicate (*n* = 3) and challenged with *Aeromonas hydrophila* (1 × 10^6^ CFU/fish) for 10 days. Different letters (a,b) indicate a significant difference (*p* < 0.05).

Cumulative survival rates of Nile tilapia fed six experimental diets and challenged with *Aeromonas hydrophila* (1 × 10^6^ CFU/fish) are presented in Figure 2. At 10 days after pathogenic bacteria injection, the cumulative survival rate of fish fed the SSE diet was significantly higher than for fish fed CON, SQSE and TSE diets (*p* < 0.05). However, fish fed the SSE diet showed no significant differences in cumulative survival rate as compared to fish fed SSEP_2_ and SSEP_4_ diets (*p* > 0.05).

## 4. Discussion

Experimental diets prepared for this research were well-accepted by tilapia, and almost no remaining feed was observed in aquaria one hour after feeding. In the present study, the results showed that the SSE, SSEP_2_ and SSEP_4_ diets could improve the growth performance compared with the control group. Interestingly, total feed intake in fish fed the SSE and SSEP_4_ diets was significantly higher than for the control diet, which could be attributed to the high palatability of the feeds ingested by the fish. This was reflected in terms the higher growth rate of those fish compared to those on other diets. Growth performance is an important factor with which to evaluate the palatability of feed ingredients. In agreement with the present study, Leal et al. [36] reported that dietary shrimp protein hydrolysate could improve the growth performance of Nile tilapia. Furthermore, Plascencia-Jatomea et al. [11] reported that 10% dietary shrimp head silage protein hydrolysate could improve the growth performance and feed utilization of Nile tilapia. From our previous studies, we found that supplementation of 2% SSE in animal or plant protein sourced diets had positive impacts on the growth and palatability of feeds in rainbow trout and pacific white shrimp [2,3,12]. Robert et al. [37,38] reported that high quality protein hydrolysates can be obtained from tilapia and shrimp wastes. Moreover, Hung [39] found that SSE may contain a large amount of free amino acids that act as a feed attractant which is collected from the shrimp industry. Research on some other species showed similar results using shrimp protein hydrolysates. For example, Khosravi et al. [40] reported that dietary shrimp hydrolysate could improve the growth performance in low fishmeal diets for red sea bream, *Pagrus major*. Similarly, Leduc et al. [10] reported that 5% dietary shrimp hydrolysate could improve growth performance, villi length and goblet cell number, while using a low-fishmeal diet in European sea bass, *Dicentrarchus labrax*. In sea bass larvae, dietary shrimp hydrolysate stimulated larval growth compared to the control group [41]. The functionality of nucleotides as low molecular weight compounds is well-known in terms of increasing diet palatability, immunity and disease resistance in fish; they ultimately enhance aquaculture [23]. Dietary supplementation of nucleotides including IMP showed an improvement in the growth performance of different fish species, such as grouper (*Epinephelus malabaricus*), rainbow trout (*Oncorhynchus mykiss)*, Atlantic salmon (*Salmo salar*) and red sea bream (*Pagrus major*) [26,27,42,43]. In the present study, dietary IMP did not seem to affect the growth performance of tilapia. In contrast to the aforementioned studies that observed the positive effects of IMP on growth performance, Zhang et al. [44] reported no significant differences in the growth performance of gibel carp (*Carassius auratus*) fed IMP. Inconsistent results for the effects of IMP on fish growth could be related to different feed formulations, culture conditions and physiological characteristics of fish species. Furthermore, in the present study, dietary supplementations of high IMP (4%) together with SSE significantly increased the feed intake in fish compared to the control diet, which endorsed the high palatability of the feeds for juvenile Nile tilapia. In agreement with our study, Hossain et al. [27] found high feed intake in supplementing IMP in the diets for red sea bream. However, contrary to the present study, Kader et al. [30] could not find any positive effect of IMP on feed intake in juvenile Nile tilapia.

In this study, the whole body proximate composition of Nile tilapia was not significantly affected by SSE. Khosravi et al. [45] reported that dietary shrimp hydrolysates did not affect whole body proximate composition in olive flounder, *Paralichtys olivaceus*. Gisbert et al. [46] also reported that dietary shrimp protein hydrolysates did not affect whole body proximate composition in European sea bass, *Dicentrarchus labrax*. According to these findings and the results of the present study, dietary shrimp protein hydrolysates might not have influenced the whole-body composition of Nile tilapia because the administered level was not very high (2% of the diet), and the proximate compositions of diets were almost the same.

Hematological parameters are useful indicators for evaluating the physiological parameters and health status of fish [47]. Based on the results of this study, SSE did not affect the alanine aminotransferase (ALT) or aspartate aminotransferase (AST) of Nile tilapia. Similarly, Khosravi et al. [40] reported that dietary shrimp hydrolysates could not affect AST and ALT in red sea bream. The present study also demonstrated no significant differences among dietary treatments regarding serum glucose (GLU) and total cholesterol (TCHO), which is in agreement with the study conducted by Khosravi et al. [48].

Fishery by-product protein hydrolysates have been reported to improve antimicrobial [38,49], antioxidant [50] or antihypertensive activities [51]. Non-specific immune responses such as superoxide dismutase, myeloperoxidase and lysozyme activity are useful parameters for evaluating health status in fish [52]. In the present research, fish fed the SSE and SSEP_4_ diets improved serum MPO and SOD activities compared to fish fed the CON, SSEP_2_ and SQSE diets. Additionally, it is worth mentioning that SQSE and TSE diets resulted in higher SOD and MPO activity as compared to the CON diet. Khosravi et al. [40] reported that dietary shrimp hydrolysates could improve SOD activity in low-fishmeal diets for red sea bream, *Pargus major*. However, in the present study, SSEP_2_ resulted in significantly lower SOD, MPO and lysozyme activity compared to SSE and SSEP_4_. This could have been caused by an error during the sampling or analysis, and therefore, further considerations are required in this regard. However, the lysozyme activity of fish fed the SSE and SSEP_4_ diets showed no significant differences with fish fed the CON diet. Gisbert et al. [46] reported that having 5% of a diet be dietary shrimp protein hydrolysate could improve serum lysozyme activity in European sea bass, *Dicentrarchus labrax*. The inconstancy in results of the present study and previous findings could be explained by different protein hydrolysates used, various administration dosages and target fish species.

This experiment indicated that disease resistance against *A. hydrophila* was improved by supplementation of SSE in tilapia diet. Likewise, Bui et al. [53] reported that dietary krill protein hydrolysate could improve disease resistance against *Edwardsiella tarda* in red sea bream, *Pagrus major*. In another study, Khosravi et al. [40] reported that dietary shrimp hydrolysate could improve disease resistance against *Edwardsiella tarda* in red sea bream. Additionally, Khosravi et al. [48] postulated that dietary tilapia, krill and shrimp hydrolysates can significantly enhance disease resistance against *Edwardsiella tarda* in olive flounder, *paralichthys olivaceus*. In the present study, it could be corroborated that dietary crustacean hydrolysates such as shrimp soluble extract with low molecular weight hydrolysates, could improve disease resistance in juvenile Nile tilapia.

## 5. Conclusions

Taken together, the results of the present study demonstrated that dietary shrimp soluble extract could improve growth performance, non-specific immune responses and disease resistance in juvenile Nile tilapia. Moreover, inosine monophosphate did not add further benefits to the SSE diet. Further research is warranted to better understand the effects of the additives based on nutrigenomic approaches.

## Figures and Tables

**Table 1:** Formulations and proximate compositions of six experimental diets (percentages of dry matter basis).

Ingredients (% in Diet)	Diets					
	CON	SSE	SSEP_2_	SSEP_4_	SQSE	TSE
Fishmeal (Tuna) ^1^	8.00	8.00	8.00	8.00	8.00	8.00
Soybean meal ^1^	34.60	34.00	34.00	34.00	34.00	34.00
Wheat flour ^1^	34.99	33.54	33.54	33.54	33.54	33.54
Rapeseed meal ^1^	10.00	10.00	10.00	10.00	10.00	10.00
Meat and bone meal ^1^	2.00	2.00	2.00	2.00	2.00	2.00
Poultry offal meal ^1^	2.00	2.00	2.00	2.00	2.00	2.00
Squid liver meal ^1^	2.00	2.00	2.00	2.00	2.00	2.00
Soybean oil ^1^	1.30	1.34	1.34	1.34	1.34	1.34
Fish oil ^2^	2.00	2.00	2.00	2.00	2.00	2.00
Other ^3^	3.11	3.12	3.12	3.12	3.12	3.12
SSE ^4^		2.00				
SSE + IMP ^5^ 2%			2.00			
SSE + IMP 4%				2.00		
SQSE ^6^					2.00	
TSE ^7^						2.00
Total	100	100	100	100	100	100
	**Proximate Analysis (% of Dry Matter Basis)**					
Moisture	9.42	9.90	9.84	9.22	9.82	9.08
Crude protein	33.32	32.64	32.90	33.25	32.40	32.33
Crude lipid	5.20	5.79	5.78	5.41	5.92	6.22
Crude ash	8.02	8.08	8.08	8.28	8.16	8.36

^1^ CJ Ceiljedang Co. Seoul, Korea. ^2^ The feed Co. Goyang, Korea. ^3^ Other: Mineral premix, vitamin premix, protide (nucleotide by-product) and protease (CJ Ceiljedang Co.). ^4^ SSE: Shrimp soluble extract. ^5^ IMP: Inosine monophosphate; ^6^ SQSE: Squid soluble extract. ^7^ TSE: Tilapia soluble extract.

**Table 2:** Growth performances of juvenile Nile tilapia fed six experimental diets for eight weeks ^1^.

	Diets					
	CON	SSE	SSEP_2_	SSEP_4_	SQSE	TSE
WG ^2^	254.01 ± 3.57 ^b^	264.24 ± 2.71 ^a^	264.71 ± 4.67 ^a^	264.95 ± 2.87 ^a^	253.49 ± 5.32 ^b^	256.84 ± 4.13 ^ab^
SGR ^3^	2.63 ± 0.02 ^b^	2.69 ± 0.02 ^a^	2.70 ± 0.03 ^a^	2.70 ± 0.02 ^a^	2.63 ± 0.03 ^b^	2.65 ± 0.02 ^ab^
FE ^4^	76.90 ± 0.93 ^ns^	71.41 ± 1.79	75.04 ± 3.66	74.21 ± 1.58	72.91 ± 2.19	72.62 ± 4.07
FI ^5^	354.70 ± 7.52 ^b^	397.20 ± 9.12 ^a^	380.60 ± 5.03 ^ab^	384.70 ± 7.05 ^a^	373.40 ± 6.12 ^ab^	377.80 ± 8.50 ^ab^
PER ^6^	2.09 ± 0.03 ^ns^	1.97 ± 0.05	2.06 ± 0.10	2.03 ± 0.04	2.03 ± 0.06	2.04 ± 0.11
Survival ^7^	100.00 ± 0.00 ^ns^	100.00 ± 0.00	100.00 ± 0.00	100.00 ± 0.00	98.25 ± 2.48	98.25 ± 2.48
HSI ^8^	0.69 ± 0.02 ^ns^	0.74 ± 0.06	0.72 ± 0.04	0.73 ± 0.10	0.71 ± 0.07	0.77 ± 0.09
VSI ^9^	6.68 ± 0.04 ^ns^	6.76 ± 0.01	6.64 ± 0.20	6.70 ± 0.16	6.65 ± 0.14	6.75 ± 0.09
CF ^10^	1.52 ± 0.07 ^ns^	1.55 ± 0.07	1.54 ± 0.01	1.52 ± 0.03	1.55 ± 0.01	1.54 ± 0.03

^1^ Values are mean ± SD from triplicate groups of fish (*n* = 3); values in each row with different superscripts (a,b) are significantly different (*p* < 0.05) and the values in each row with no superscripts are non-significantly (ns) different (*p* < 0.05). ^2^ Weight gain (WG; %) = (final weight -initial weight) × 100/initial weight. ^3^ Specific growth rate (SGR; %/day) = (ln final weight—ln initial weight) × 100/d. ^4^ Feed efficiency (FE; %) = wet WG (g) × 100/dry feed intake (g). ^5^ FI = total feed intake (g). ^6^ Protein efficiency ratio (PER) =wet weight gain/protein intake. ^7^ Survival (SR; %) = (total fish-dead fish) × 100/total fish. ^8^ Hepatosomatic index (HSI; %) = (liver weight/body weight) × 100. ^9^ Viscerosomatic index (VSI; %) = (visceral weight/body weight) × 100. ^10^ Condition factor (CF) = (fish weight (g)/fish length (cm)^3^) × 100.

**Table 3:** Whole body proximate composition of Nile tilapia fed six experimental diets for eight weeks (% dry matter basis) ^1^.

	Diets					
	CON	SSE	SSEP_2_	SSEP_4_	SQSE	TSE
Moisture	72.69 ± 0.35 ^ns^	71.88 ± 2.14	71.67 ± 1.37	71.03 ± 0.45	72.76 ± 0.65	72.66 ± 0.26
Crude protein	15.77 ± 0.22 ^ns^	16.20 ± 1.15	16.55 ± 0.81	16.58 ± 0.18	15.99 ± 0.40	15.87 ± 0.55
Crude Lipid	6.63 ± 0.77 ^ns^	6.29 ± 1.54	6.72 ± 0.63	7.39 ± 0.51	6.87 ± 0.67	6.36 ± 1.35
Crude Ash	4.56 ± 0.17 ^ns^	4.72 ± 0.20	5.08 ± 0.43	5.00 ± 0.32	4.77 ± 0.81	4.55 ± 0.31

^1^ Values are mean ± SD from triplicate groups of fish (*n* = 3) where the values in each row with no superscripts are non-significantly (ns) different (*p* < 0.05).

**Table 4:** Hematological parameters of Nile tilapia fed six experimental diets for eight weeks ^1^.

	Diets					
	CON	SSE	SSEP_2_	SSEP_4_	SQSE	TSE
AST ^2^	76.0 ± 4.6 ^ns^	77.0 ± 2.6	75.0 ± 2.0	75.0 ± 3.0	75.0 ± 2.0	74.7 ± 2.1
ALT ^3^	3.0 ± 1.0 ^ns^	2.0 ± 0.0	3.0 ± 1.0	2.7 ± 0.6	2.7 ± 0.6	2.7 ± 0.6
GLU ^4^	44.3 ± 2.3 ^ns^	43.0 ± 1.0	45.3 ± 1.5	44.0 ± 1.0	43.0 ± 1.0	43.0 ± 1.0
TCHO ^5^	224.3 ± 0.6 ^ns^	224.0 ± 1.0	225.3 ± 1.5	224.3 ± 0.6	224.0 ± 1.7	225.3 ± 1.5

^1^ Values are mean ± SD from triplicate groups of fish (*n* = 3) where the values in each row with no superscripts are non-significantly (ns) different (*p* < 0.05). ^2^ Aspartate aminotransferase, AST (U/L). ^3^ Alanine aminotransferase, ALT (U/L). ^4^ Glucose, GLU (mg/dL). ^5^ Total-Cholesterol, TCHO (mg/dL).

## Data Availability

The data presented in this study are available on request from the corresponding author.

## References

[1] FAO (2021). Food and Agriculture Organization FISHSTAT Plus.

[2] Moniruzzaman M., Bae J.H., Won S.H., Cho S.J., Chang K.H., Bai S.C. (2018). Evaluation of solid-state fermented protein concentrates as a fish meal replacer in the diets of juvenile rainbow trout, *Oncorhynchus mykiss*. Aquacult. Nutr..

[3] Moniruzzaman M., Damusaru J.H., Won S.H., Cho S.J., Chang K.H., Bai S.C. (2020). Effects of partial replacement of dietary fish meal by bioprocessed plant protein concentrates on growth performance, hematology, nutrient digestibility and digestive enzyme activities in juvenile Pacific white shrimp, *Litopenaeus Vannamei*. J. Sci. Food Agric..

[4] Herath S.S., Haga Y., Satoh S. (2016). Potential use of corn co-products in fishmeal-free diets for juvenile Nile tilapia *Oreochromis niloticus*. Fish. Sci..

[5] Koch J.F., Rawles S.D., Webster C.D., Cummins V., Kobayashi Y., Thompson K.R., Gannam A.L., Twibell R.G., Hyde N.M. (2016). Optimizing fish meal-free commercial diets for Nile tilapia, *Oreochromis niloticus*. Aquaculture.

[6] Romano N., Kumar V., Yang G., Kajbaf K., Rubio M.B., Overturf K., Brezas A., Hardy R. (2020). Bile acid metabolism in fish: Disturbances caused by fishmeal alternatives and some mitigating effects from dietary bile inclusions. Rev. Aquacult..

[7] Toften H., Jobling M. (1997). Feed intake and growth of Atlantic salmon, *Salmo salar* L., fed diets supplemented with oxytetracycline and squid extract. Aquacult. Nutr..

[8] Chalamaiah M., Kumar B.D., Hemalatha R., Jyothirmayi T. (2012). Fish protein hydrolysates: Proximate composition, amino acid composition, antioxidant activities and applications: A review. Food Chem..

[9] Gildberg A. (1992). Enzymic processing of marine raw materials. Process Biochem..

[10] Leduc A., Zatylny-Gaudin C., Khosravi M., Corre E., Le Corguille G., Castel H., Lefevre-Scelles A., Fournier V., Gisbert E., Andree K.B. (2018). Dietary aquaculture by-product hydrolysates: Impact on the transcriptomic response of the intestinal mucosa of European seabass (*Dicentrarchus labrax*) fed low fish meal diets. BMC Genom..

[11] Plascencia-Jatomea M., Olvera-Novoa M.A., Arredondo-Figueroa J.L., Hall G.M., Shirai K. (2002). Feasibility of fishmeal replacement by shrimp head silage protein hydrolysate in Nile tilapia (*Oreochromis niloticus* L.) diets. J. Sci. Food Agric..

[12] Jo H., Lee S., Yun H., Hong J.W., Moniruzzaman M., Bai S.C., Park G., Chee S., Jeon T.E. (2017). Evaluation of dietary fishmeal analogue with addition of shrimp soluble extract on growth and nonspecific immune response of rainbow trout, *Oncorhynchus mykiss*. J. World Aquacult. Soc..

[13] Kolkovski S., Tandler A. (2000). The use of squid protein hydrolysate as a protein source in microdiets for gilthead seabream *Sparus aurata* larvae. Aquacult. Nutr..

[14] Foh M.B., Kamara M.T., Amadou I., Foh B.M., Xia W. (2011). Chemical and physicochemical properties of tilapia (*Oreochromis niloticus*) fish protein hydrolysate and concentrate. Int. J. Biol. Chem..

[15] Fan J., He J., Zhuang Y., Sun L. (2012). Purification and identification of antioxidant peptides from enzymatic hydrolysates of tilapia (*Oreochromis niloticus*) frame protein. Molecules.

[16] Jobling M., Koskela J., Savolainen R. (1998). Influence of dietary fat level and increased adiposity on growth and fat deposition in rainbow trout, *Oncorhynchus mykiss* (Walbaum). Aquacult. Res..

[17] Lian P., Lee C.M., Park E. (2005). Characterization of Squid-Processing Byproduct Hydrolysate and Its Potential as Aquaculture Feed Ingredient. J. Agric. Food Chem..

[18] Gildberg A., Stenberg E. (2001). A new process for advanced utilisation of shrimp waste. Process Biochem..

[19] Aksnes A., Hope B., Jönsson E., Björnsson B.T., Albrektsen S. (2006). Size-fractionated fish hydrolysate as feed ingredient for rainbow trout (*Oncorhynchus mykiss*) fed high plant protein diets. I: Growth, growth regulation and feed utilization. Aquaculture.

[20] El-Sayed A.F. (2020). Tilapia Culture.

[21] Abdel-Tawwab M., Abdel-Rahman A.M., Ismael N.E. (2008). Evaluation of commercial live bakers’ yeast, Saccharomyces cerevisiae as a growth and immunity promoter for Fry Nile tilapia, *Oreochromis niloticus* (L.) challenged in situ with *Aeromonas hydrophila*. Aquaculture.

[22] Simon C.J., Blyth D., Ahmad F.N., Suri S. (2019). Microbial biomass (Novacq™) stimulates feeding and improves the growth performance on extruded low to zero-fishmeal diets in tilapia (GIFT strain). Aquaculture.

[23] Li P., Gatlin D.M. (2006). Nucleotide nutrition in fish: Current knowledge and future applications. Aquaculture.

[24] Carver J.D. (1994). Dietary nucleotides: Cellular immune, intestinal and hepatic system effects. J. Nutr..

[25] Haskó G., Kuhel D.G., Németh Z.H., Mabley J.G., Stachlewitz R.F., Virág L., Lohinai Z., Southan G.J., Salzman A.L., Szabó C. (2000). Inosine inhibits inflammatory cytokine production by a posttranscriptional mechanism and protects against endotoxin-induced shock. J. Immunol..

[26] Lin Y.H., Wang H., Shiau S.Y. (2009). Dietary nucleotide supplementation enhances growth and immune responses of grouper, *Epinephelus malabaricus*. Aquacult. Nutr..

[27] Hossain M.S., Koshio S., Ishikawa M., Yokoyama S., Sony N.M., Ono S., Fujieda T. (2016). Comparison of the effects of inosine and inosine monophosphate on growth, immune response, stress resistance and gut morphology of juvenile red sea bream, *Pagrus major*. Aquaculture.

[28] Song J.W., Lim S.J., Lee K.J. (2012). Effects of dietary supplementation of inosine monophosphate on growth performance, innate immunity and disease resistance of olive flounder (*Paralichthys olivaceus*). Fish Shellfish Immunol..

[29] Asaduzzaman M., Ikeda D., Abol-Munafi A.B., Bulbul M., Ali M.E., Kinoshita S., Watabe S., Kader M.A. (2017). Dietary supplementation of inosine monophosphate promotes cellular growth of muscle and upregulates growth-related gene expression in Nile tilapia *Oreochromis niloticus*. Aquaculture.

[30] Kader M.A., Bulbul M., Abol-Munafi A.B., Asaduzzaman M., Mian S., Noordin N.B.M., Ali M.E., Hossain M.S., Koshio S. (2018). Modulation of growth performance, immunological responses and disease resistance of juvenile Nile tilapia (*Oreochromis niloticus*) (Linnaeus, 1758) by supplementing dietary inosine monophosphate. Aquacult. Rep..

[31] Hamidoghli A., Won S., Farris N.W., Bae J., Choi W., Yun H., Bai S.C. (2020). Solid state fermented plant protein sources as fish meal replacers in whiteleg shrimp *Litopaeneus vannamei*. Anim. Feed Sci. Technol..

[32] AOAC (2005). Official Methods of Analysis of AOAC International.

[33] Hultmark D., Steiner H., Rasmuson T., Boman H.G. (1980). Insect immunity. Purification and properties of three inducible bactericidal proteins from hemolymph of immunized pupae of *Hyalophora cecropia*. Eur. J. Biochem..

[34] Quade M.J., Roth J.A. (1997). A rapid, direct assay to measure degranulation of bovine neutrophil primary granules. Vet. Immunol. Immunopath..

[35] Hasan M.T., Jang W.J., Lee B.J., Kim K.W., Hur S.W., Lim S.G., Bai S.C., Kong I.S. (2019). Heat-killed Bacillus sp. SJ-10 probiotic acts as a growth and humoral innate immunity response enhancer in olive flounder (*Paralichthys olivaceus*). Fish Shellfish Immunol..

[36] Leal A.L.G., de Castro P.F., de Lima J.P.V., de Souza Correia E., de Souza Bezerra R. (2010). Use of shrimp protein hydrolysate in Nile tilapia (*Oreochromis niloticus*, L.) feeds. Aquacult. Int..

[37] Robert M., Zatylny-Gaudin C., Fournier V., Corre E., Le Corguille G., Bernay B., Henry J. (2014). Transcriptomic and peptidomic analysis of protein hydrolysates from the white shrimp (L. *vannamei*). J. Biotechnol..

[38] Robert M., Zatylny-Gaudin C., Fournier V., Corre E., Le Corguille G., Bernay B., Henry J. (2015). Molecular characterization of peptide fractions of a Tilapia (*Oreochromis niloticus*) by-product hydrolysate and in vitro evaluation of antibacterial activity. Process Biochem..

[39] Hung L.T. (2014). Shrimp soluble extract-novel feed attractant for aquaculture. Glob. Aquac. Advocate.

[40] Khosravi S., Rahimnejad S., Herault M., Fournier V., Lee C.R., Bui H.T.D., Jeong J.B., Lee K.J. (2015). Effects of protein hydrolysates supplementation in low fish meal diets on growth performance, innate immunity and disease resistance of red sea bream *Pagrus major*. Fish Shellfish Immunol..

[41] Delcroix J., Gatesoupe F.J., Desbruyeres E., Huelvan C., Le Delliou H., Le Gall M.M., Quazuguel P., Mazurais D., Zambonino-Infante J.L. (2015). The effects of dietary marine protein hydrolysates on the development of sea bass larvae, *Dicentrarchus labrax*, and associated microbiota. Aquacult. Nutr..

[42] Burrells C., Williams P.D., Southgate P.J., Wadsworth S.L. (2001). Dietary nucleotides: A novel supplement in fish feeds: 2. Effects on vaccination, salt water transfer, growth rates and physiology of Atlantic salmon (*Salmo salar* L.). Aquaculture.

[43] Tahmasebi-Kohyani A., Keyvanshokooh S., Nematollahi A., Mahmoudi N., Pasha-Zanoosi H. (2011). Dietary administration of nucleotides to enhance growth, humoral immune responses, and disease resistance of the rainbow trout (*Oncorhynchus mykiss*) fingerlings. Fish Shellfish Immunol..

[44] Zhang P., Fu L., Liu H., Huda N.U., Zhu X., Han D., Jin J., Yang Y., Kim Y.S., Xie S. (2019). Effects of inosine 5′-monophosphate supplementation in high fishmeal and high soybean diets on growth, immune-related gene expression in gibel carp (*Carassius auratus gibelio* var. CAS Ⅲ), and its challenge against *Aeromonas hydrophila* infection. Fish Shellfish Immunol..

[45] Khosravi S., Bui H.T.D., Herault M., Fournier V., Kim K.D., Kim K.W., Lee K.J. (2018). Supplementation of protein hydrolysates to a low-fishmeal diet improves growth and health status of Juvenile Olive Flounder, *Paralichthys olivaceus*. J. World Aquacult. Soc..

[46] Gisbert E., Fournier V., Solovyev M., Skalli A., Andree K.B. (2018). Diets containing shrimp protein hydrolysates provided protection to European sea bass (*Dicentrarchus labrax*) affected by a *Vibrio pelagius* natural infection outbreak. Aquaculture.

[47] Maita M. (2007). Fish Health Assessment. Dietary Supplements for the Health and Quality of Cultured Fish.

[48] Khosravi S., Bui H.T.D., Rahimnejad S., Herault M., Fournier V., Jeong J.B., Lee K.J. (2015). Effect of dietary hydrolysate supplementation on growth performance, non-specific immune response and disease resistance of olive flounder (*Paralichthys olivaceus*) challenged with *Edwardsiella tarda*. Aquac. Nutr..

[49] Sila A., Nedjar-Arroume N., Hedhili K., Chataigne G., Balti R., Nasri M., Dhulster P., Bougatef A. (2014). Antibacterial peptides from barbel muscle protein hydrolysates: Activity against some pathogenic bacteria. LWT Food Sci. Technol..

[50] García-Moreno P.J., Batista I., Pires C., Bandarra N.M., Espejo-Carpio F.J., Guadix A., Guadix E.M. (2014). Antioxidant activity of protein hydrolysates obtained from discarded Mediterranean fish species. Food Res. Int..

[51] Ktari N., Nasri R., Mnafgui K., Hamden K., Belguith O., Boudaouara T., El Feki A., Nasri M. (2014). Antioxidative and ACE inhibitory activities of protein hydrolysates from zebra blenny (*Salaria basilisca*) in alloxan-induced diabetic rats. Process Biochem..

[52] Ainsworth A.J., Dexiang C., Waterstrat P.R. (1991). Changes in peripheral blood leukocyte percentages and function of neutrophils in stressed channel catfish. J. Aquat. Anim. Health.

[53] Bui H.T.D., Khosravi S., Fournier V., Herault M., Lee K.J. (2014). Growth performance, feed utilization, innate immunity, digestibility and disease resistance of juvenile red seabream (*Pagrus major*) fed diets supplemented with protein hydrolysates. Aquaculture.

